# Supplementary material for: From sequence to enzyme mechanism using multi-label machine learning
Source: BMC Bioinformatics. 2014 May 19;15:150. doi: 10.1186/1471-2105-15-150 (PMC4229970; doi:10.1186/1471-2105-15-150)
Supplement: Additional file 2 — Java code of ml2db. Additional file ml2db_code.tar.gz contains the Java source code to run the multi-label machine learning experiments and save the results to database. The code’s Javadoc is included. [file 1471-2105-15-150-S2.zip › additional file 2/ml2db/ml2db/doc/mulan/experiments/Experiment.html]

Experiment


JavaScript is disabled on your browser.


- Overview
- Package
- Class
- Use
- Tree
- Deprecated
- Index
- Help

- Prev Class
- Next Class

- Frames
- No Frames

- All Classes

- Summary:
- Nested |
- Field |
- Constr |
- Method

- Detail:
- Field |
- Constr |
- Method


mulan.experiments

## Class Experiment

- java.lang.Object
- - mulan.experiments.Experiment

- All Implemented Interfaces:
  :   weka.core.TechnicalInformationHandler

  Direct Known Subclasses:
  :   ICDM08EnsembleOfPrunedSets, ICTAI2010, MachineLearning09IBLR, PatternRecognition07MLkNN

  ---

    

  ```
  public abstract class Experiment
  extends java.lang.Object
  implements weka.core.TechnicalInformationHandler
  ```

  Abstract base class for all experiments

  Version:
  :   2010.12.27

  Author:
  :   Eleftherios Spyromitros-Xioufis, Grigorios Tsoumakas

- - ### Constructor Summary

    Constructors

    Constructor and Description

    `Experiment()`
  - ### Method Summary

    Methods

    | Modifier and Type | Method and Description |
    | --- | --- |
    | `abstract weka.core.TechnicalInformation` | `getTechnicalInformation()` Returns an instance of a TechnicalInformation object, containing detailed information about the technical background of this class, e.g., paper reference or book this class is based on. |

    - ### Methods inherited from class java.lang.Object

      `equals, getClass, hashCode, notify, notifyAll, toString, wait, wait, wait`

- - ### Constructor Detail


    - #### Experiment

      ```
      public Experiment()
      ```
  - ### Method Detail


    - #### getTechnicalInformation

      ```
      public abstract weka.core.TechnicalInformation getTechnicalInformation()
      ```

      Returns an instance of a TechnicalInformation object, containing
      detailed information about the technical background of this class,
      e.g., paper reference or book this class is based on.

      **Specified by:**
      :   `getTechnicalInformation` in interface `weka.core.TechnicalInformationHandler`

      Returns:
      :   the technical information about this class


- Overview
- Package
- Class
- Use
- Tree
- Deprecated
- Index
- Help

- Prev Class
- Next Class

- Frames
- No Frames

- All Classes

- Summary:
- Nested |
- Field |
- Constr |
- Method

- Detail:
- Field |
- Constr |
- Method
